# Supplementary material for: Frozen sound: An ultra-low frequency and ultra-broadband non-reciprocal acoustic absorber
Source: Nat Commun. 2023 Jul 7;14:4028. doi: 10.1038/s41467-023-39727-4 (PMC10329010; doi:10.1038/s41467-023-39727-4)
Supplement: Supplementary file 1 — Supplementary Information [file 41467_2023_39727_MOESM1_ESM.pdf]

Supplementary information for Frozen sound: An ultra-low frequency and ultra-broadband non-reciprocal acoustic absorber

Anis Maddi,[1] Come Olivier,[1] Gaelle Poignand,[1] Guillaume Penelet,[1] Vincent Pagneux,[1] and Yves Auregan[1]
Laboratoire d'Acoustique de l'Université du Mans (LAUM), UMR 6613, Institut d'Acoustique - Graduate School (IA-GS), CNRS, Le Mans Université, France

SUPPLEMENTARY METHODS 1. TRANSFER MATRIX OF A THERMOACOUSTIC CELL

This section briefly describes the method allowing to compute the transfer matrix of a thermoacoustic cell, which itself can be represented in terms of a block diagram connecting various two-ports as described in Fig.1. Each two-port can be described by means of a transfer matrix, and the considered two-port can be either an empty duct (e.g. the Thermal Buffer Tube), or a duct filled with a medium made of many adjacent channels, characterized by a global porosity and the size of a single pore.

The transfer matrix can be calculated in the frame of the linear thermoacoustic theory[1,2], which describes the lossy propagation of plane waves through ducts submitted to an axial temperature gradient as:

$$\frac{dp}{dx} = -\frac{i\omega\rho_m}{S\phi\,(1-f_\nu)}u,$$
$$\frac{du}{dx} = -\frac{i\omega S\phi}{\gamma p_m}\left[1+(\gamma-1)f_\kappa\right]p + \frac{f_\kappa - f_\nu}{(1-f_\nu)\,(1-\sigma)}\frac{1}{T_m}\frac{dT_m}{dx}u, \quad (1)$$

where p and u respectively stand for the complex amplitudes of acoustic pressure and volume velocity at some position x. In Eqs (1) the parameters $\rho_m(x)$, p_m and $T_m(x)$ respectively stand for the mean (time-averaged) density, the mean pressure and the mean axial temperature, γ is the specific heat ratio of the fluid, S corresponds to the cross-sectional area of the duct, and $\sigma = \nu_0/\kappa_0$ is the Prandtl number of the fluid (ν_0 and κ_0 are the kinematic viscosity and the thermal diffusivity of the fluid at room temperature T_0). These equations hold either for an empty duct or for a duct filled with a porous medium having a porosity ϕ ($\phi < 1$ in the stack or in the heat-exchangers, and $\phi = 1$ elsewhere). The thermoacoustic process is described by means of the thermoviscous functions f_κ and f_ν, which depend on the geometry and on the dimensions of the channel. For instance, in the case of a pile of cylindrical pores, the viscothermal functions are given by

$$f_{\nu,\kappa} = \frac{2}{(1-i)r/\delta_{\nu,\kappa}}\frac{J_1\left[(1-i)r/\delta_{\nu,\kappa}\right]}{J_0\left[(1-i)r/\delta_{\nu,\kappa}\right]}, \quad (2)$$

where J_m is the Bessel function of order m, and r is the radius of a single pore. The Thermal Buffer Tube is treated as a single rectangular channel, while the stack and the heat exchangers are treated as a pile of adjacent cylindrical channels (see Fig. 1).

A discrete version of Eqs.(1) is used to solve the problem under consideration, and the acoustic propagation through a short portion of length $\Delta x_n = x_{n+1} - x_n$ (with Δx_n much shorter than the wavelength $2\pi c/\omega$, where $c = \sqrt{\gamma p_m/\rho_m}$ is the speed of sound), is described by means of a forward Euler finite-difference scheme, i.e. by setting $(d\xi/dx) \approx (\xi_{n+1} - \xi_n)/\Delta x_n$ where ξ either stands for p, u or T_m. The discrete version of the propagation equations therefore writes as:

$$p_{n+1} = p_n - \frac{i\omega\rho_n\Delta x_n}{\phi S}\frac{1}{1-f_{\nu_n}}u_n, \quad (3)$$
$$u_{n+1} = -\frac{i\omega\phi S\Delta x_n}{\gamma p_m}\left[1+(\gamma-1)f_{\kappa_n}\right]p_n + \left[1+\frac{f_{\kappa_n}-f_{\nu_n}}{(1-f_{\nu_n})(1-\sigma)}\frac{T_{n+1}-T_n}{T_n}\right]u_n, \quad (4)$$

where p_n, u_n and T_n stand for the pressure, the volume velocity and the mean temperature at position $x = x_n$, and where $\rho_n = \rho_0 T_0/T_n$ stands for the fluid density at temperature T_n. Note that the variations with temperature of the viscothermal functions $f_{\nu_n,\kappa_n} = f_{\nu,\kappa}(\omega, T_n)$, due to the variations of ν and κ with T_n, are taken into account using the empirical laws $\nu_n = \nu_0(T_n/T_0)^{1+\beta}$ and $\kappa_n = \kappa_0(T_n/T_0)^{1+\beta}$ (with $\beta = 0.7$ for air). If the temperature distribution is assigned at any position x along the thermoacoustic cell, and if each sub-component of a TA cell (stack, heat-exchangers, TBT) is itself discretized as a large number of elements of length Δx_n, then Eqs. (3)-(4) can be used to compute the transfer matrix $\mathbf{M}$ of each of the sub-components shown in Fig. 1.As a result, As a result, once each two-port is characterized in terms of a transfer matrix, it is straightforward to calculate the transfer matrix of a thermoacoustic cell (or two thermoacoustic cells in series).

SUPPLEMENTARY METHODS 2. SIMPLIFIED T-MATRIX OF A SHORT STACK WITH THIN PORES

Next, attention is focused on the stack, which has a length L_s and is (assumed to be) made up of many channels with radius r_s. If we assume that the typical size of a pore is much smaller than the viscothermal boundary layer thicknesses, $r_s \ll \delta_{\kappa,\nu}$, the viscothermal functions $f_{\kappa,\nu}$ at some position x_n in the stack can be approximated to lowest order as

$$f_{\kappa,n} \approx 1 - i\omega\Theta_n,$$
$$f_{\nu,n} \approx 1 - i\omega\Theta_n/\sigma,$$

where $\Theta_n = \frac{r_s^2(T_0/T_n)^{1+\beta}}{8\kappa_0}$.

Moreover, if the assumption of a short stack (i.e, $\omega L_s/c \ll 1$) is made such that only a single element is kept for the spatial discretization ($\Delta x \sim L_s$), then this leads to the simplification of Eqs.(3-4) to

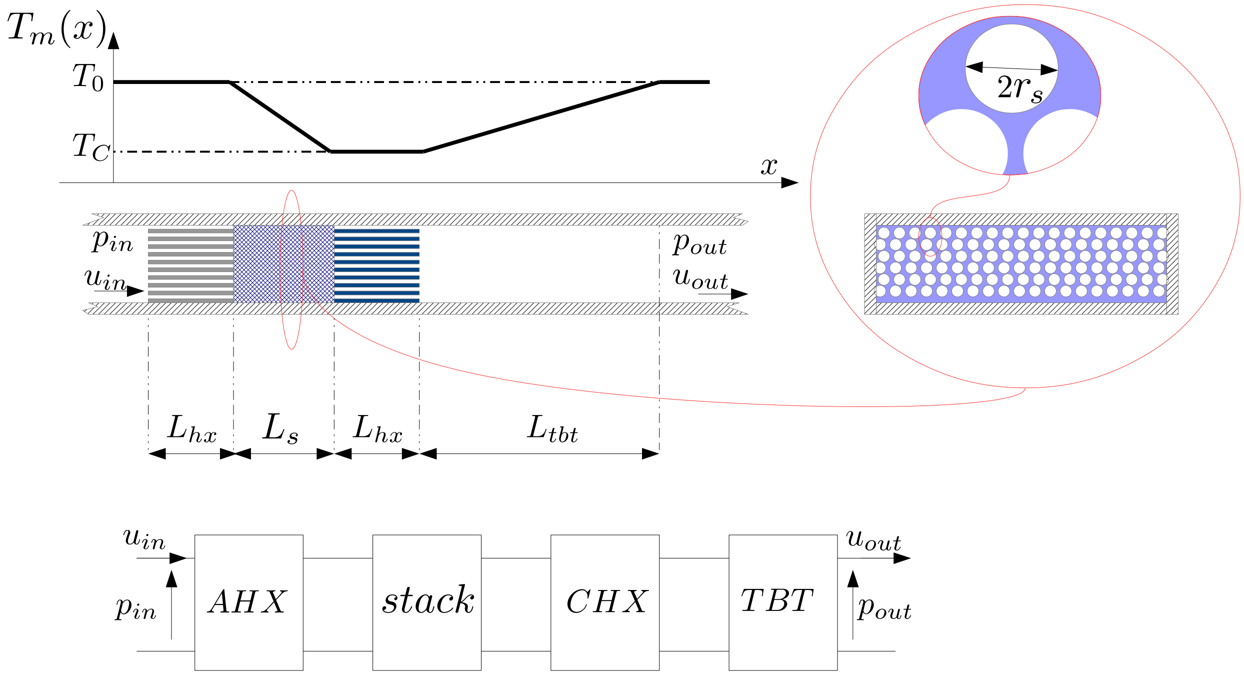

FIG. 1: Sketch of a thermoacoustic cell and its corresponding block-diagram (Ambient Heat eXchanger, stack, Cold Heat eXchanger, Thermal Buffer Tube).

$$\mathbf{M}_{reg} \approx \begin{pmatrix} 1 & -\frac{i\omega\rho_{m,0}L_s}{S\phi\left(1-f_{\nu,0}\right)} \\ -\frac{i\omega S\phi L_s\left[1+(\gamma-1)f_{\kappa,0}\right]}{\gamma p_m} & 1+\frac{f_{\kappa,0}-f_{\nu,0}}{\left(1-f_{\nu,0}\right)\left(1-\sigma_0\right)}\frac{\Delta T}{T_0} \end{pmatrix} \quad (5)$$

Finally, combining both of the previous assumptions, namely $\omega L_r/c \ll 1$ and $r_r \ll \delta_{\kappa,\nu}$, leads after simplifications to the following transfer matrix of the stack :

$$\mathbf{M}_s \approx \begin{pmatrix} 1 & -\overbrace{\frac{8\rho_{m,0}\nu_{m,0}L_s}{\phi S r_s^2}}^{R_\nu} \\ 0 & \frac{T_C}{T_0} \end{pmatrix}. \quad (6)$$

where R_ν represents the viscous resistance of the stack, which depends on both the stack geometry and the thermo-physical properties of the fluid. Furthermore, the resistivity parameter R used in the main article, is a normalized version of this viscous resistance, such that $R = R_\nu/Z$.

SUPPLEMENTARY METHODS 3. EXPERIMENTAL SETUP

The experimental setup of the liquid nitrogen broadband absorber is shown in Fig.2. The complete setup consists of two long ducts (equipped with microphones) connected to the absorber. The cross-sectional area of the waveguide is $S = 5\text{cm}\times 1\text{cm}$.

The absorber consists of either one cell or two cells in series, depending on the chosen configuration. Each cell is equipped with a stack of length $L_s = 1.6$ cm, made of stacked stainless steel wiremeshes, with an estimated porosity $\phi = 0.8$ and an estimated pore radius $r_s = 72\mu\text{m}$. A cold and an ambient heat exchanger are attached to each side of the stack: they both consist of a honeycombed aluminum material with a length $L = 1.5$cm, a porosity $\phi = 0.945$, and a pore radius $r = 450\mu\text{m}$. Heat can be supplied or removed from the system through those heat exchangers, using heat sources which are in thermal contact with the heat exchangers through the waveguide wall (1 mm in thickness). The cold source consists of a pair of insulated aluminum reservoirs of inner volume $V = 52\text{cm}^3$ filled with liquid nitrogen. During operation and due to the quick evaporation of liquid nitrogen, more liquid is regularly added to maintain the reservoirs full. The hot source consists of cartridge heaters inserted in solid aluminum blocks which are placed at the heat exchanger location on either side of the waveguide. Close to the hot source, a thermocouple was placed at the external face of the duct: it was used to monitor the hot temperature T_0 and to make so that this temperature stays close to the room temperature by adjusting the power $\mathscr{P}$ supplied to the heater. The last element of the thermoacoustic cell is the thermal buffer tube of length $L_{TBT} = 3$cm along

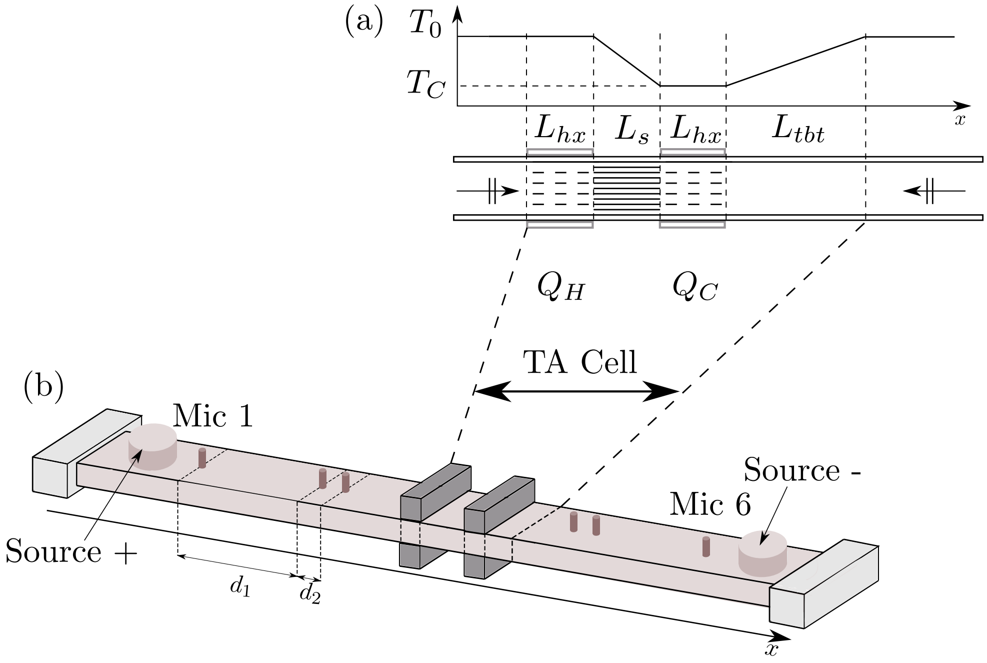

FIG. 2: (a) Representation of the unit cell and its temperature distribution. (b) Sketch of the experimental apparatus for the measurement of the absorber's transfer and scattering matrices.

which the temperature gradually increases up to room temperature. As a result, the total length of a single thermoacoustic cell is $L_{cell} = 7.1\,\text{cm}$.

A two-source method is used for the measurement of the system's transfer and scattering matrices[3]. Three microphones (Bruel and Kjær 4136 with Nexus amplifier 2690, Denmark) are flush mounted on each side of the measurement apparatus, thus forming two pairs of microphones for measurements in a specific frequency range. The first pair separated by a distance $d_1 + d_2 = 75$cm allows measurement in the lower frequency range $10\,\text{Hz} < f < 220\,\text{Hz}$, while the second one separated by the distance $d_2 = 10$cm allows measurements in the higher frequency range $200\,\text{Hz} < f < 1000\,\text{Hz}$. Note that a relative calibration of the microphones was performed beforehand using a small cavity coupler, so as to minimize measurement errors[4]. The accuracy of the multiple microphones technique was also validated from the measurement of the transfer matrix of an empty duct (3 meters in length), and it was checked that the measured values of the T-matrix coefficients were matching the theoretical ones.

Each end of the measurement apparatus is connected to an anechoic termination, made with a small cavity and a layer of stacked wiremeshes (see Ref. 5 for more details on the design of such termination). The reflection coefficient of those terminations is sufficiently small to avoid any instability that may occur (i.e., onset of self-sustained thermoacoustic oscillations).

Finally, two acoustic sources are connected to the waveguide through a side-branch and a small duct. Each source consists of a moving coil loudspeaker (Visaton FRS 8) mounted in a enclosure, which allows to excite the system at low amplitude (below 10 Pa) using a harmonic signal with a step by step increasing frequency. In the following, the excitation frequencies are far below the cut-off frequency[6] of the waveguide, estimated at $f_{co} = 3400$Hz, such that only plane waves are propagating along the duct.

SUPPLEMENTARY DISCUSSION 1. ON THE IMPACT OF THE SHAPE OF THE TEMPERATURE DISTRIBUTION

As the shape of the temperature distribution within the TA cell cannot be measured, we checked numerically that it does not strongly impact the operation of the system in the low frequency range. The results are presented in Fig.3, where we have calculated both α^+ and the coefficient T_{22} of the T-matrix of the TA cell (this coefficient is supposed to tend towards $\eta = T_C/T_0$ at low-frequencies) for three different shapes of the axial temperature distribution in the stack. The results show that for different temperature profiles and under the above stated conditions, the shape of the temperature distribution will not have a significant impact on the η parameter provided that the temperature difference remains the same (See Fig 3.a). However, the viscous resistance, as opposed to the parameter η, will be affected by the temperature profile (since the viscosity of the fluid depends on temperature) and this may improve (or decrease) the absorption depending on how close (or far) this value gets in comparison to the optimal viscous resistance (as shown in Fig 3.b).

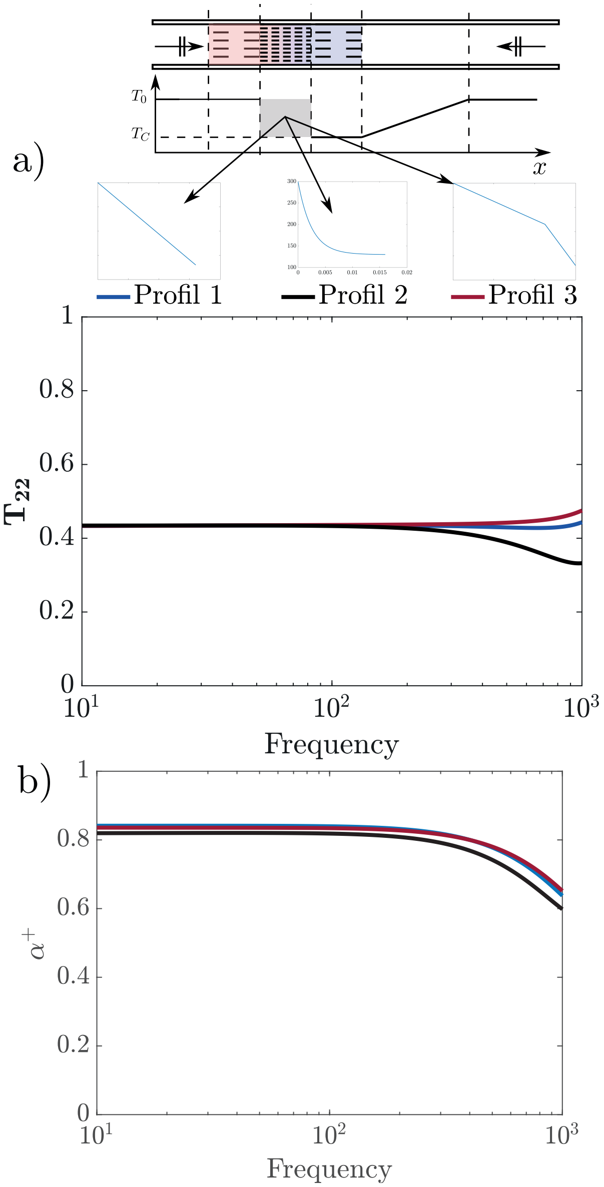

FIG. 3: Impact of the temperature gradient on the parameter η and the absorption coefficient α. (a) Coefficient $\mathbf{T_{22}}$ of the transfer matrix for three temperature profiles. This coefficient is equal to the parameter η at low frequencies. (b) Absorption coefficient α as function of the frequency, for 3 different temperature profiles.

SUPPLEMENTARY DISCUSSION 2. EFFECT OR RIGID BACKING

Although it falls out of the scope of this study where a trasnmission problem is considered, a question which may arise regarding the efficiency of the proposed non-reciprocal absorber is that of the impact a rigid backing which would be placed just behind the absorber. Measurements were not performed for such a configuration, but they can be used to predict the absorption of an incident wave by a TA cell and a rigid backing which imposes a vanishing velocity at the output of the absorber. The results obtained are presented in Fig. 4 for the case of a single TA cell. The absorption due to the TA cell equipped with a rigid backing are presented with blue or black open circle markers (depending on if cooling is applied or not) while the results for a transmission problem are provided with filled circle markers as a reminder. The main conclusion that can be drawn is that the broadband absorption at ultra-low frequency disappears if a rigid backing is placed behind the TA-cell, no matter if cooling is applied or not. Such a decrease of the absorption coefficient in the low frequency range is expected, as it is for the simpler case of ultrathin meshgrids which cannot act as a viscous resistance if they are placed too close to a rigid wall. It is worth noting that if a rigid backing is placed behind the TA-cell, there exists a frequency range where a very high absorption can be achieved (especially if cooling is applied).

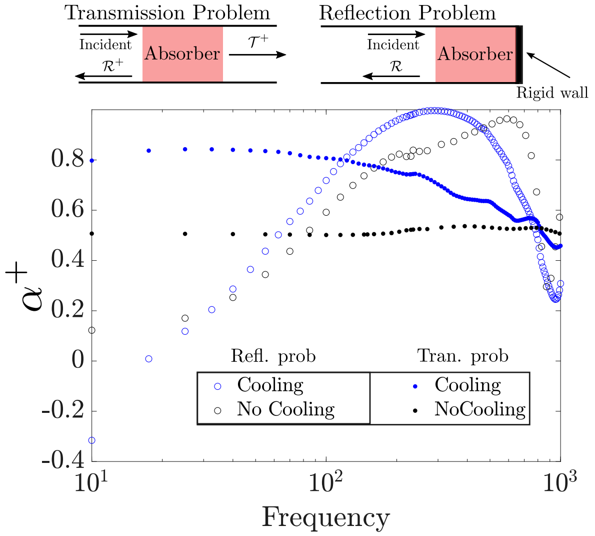

FIG. 4: Absorption coefficient α^+ for a one-cell configuration as a function of the frequency, depending on if a rigid wall is placed just behind the TA-cell (where $\alpha^+ = 1 - |\mathscr{R}^+ + \mathscr{T}^+\mathscr{T}^-(1+\sum_{n=1}^{\infty}(R^-)^n)|^2$ for a reflection problem) or if there is no rigid backing (transmission problem, where $\alpha^+ = 1 - |\mathscr{T}^+|^2 - |\mathscr{R}^+|^2$). Blue markers refer to the results obtained with cooling, while black markers refer to the results obtained without cooling, and the results are obtained from the measurements of the scattering matrix for one-cell.

SUPPLEMENTARY DISCUSSION 3. EXPERIMENTAL TRANSFER MATRIX OF A TA CELL

In addition to the absorption coefficient and the scattering matrix coefficients presented in the main document, the experimentally obtained coefficients of the transfer matrix are presented in Fig.5 as functions of the frequency for the case of a single thermoacoustic cell submitted to cooling with liquid nitrogen. The experimental data can notably be used to check the validity of Eq.(6) in the low-frequency limit.

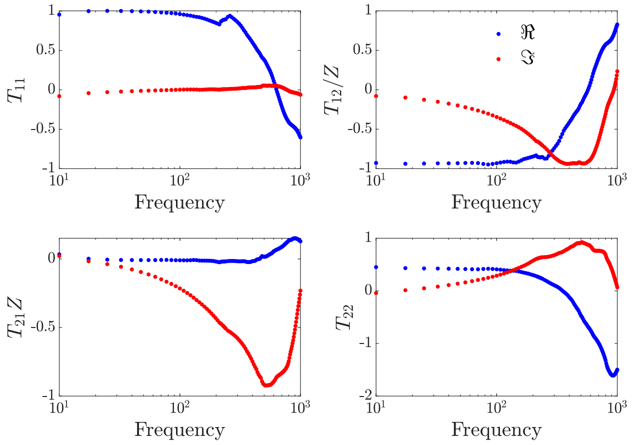

FIG. 5: Coefficients of the transfer matrix for a single thermoacoustic cell submitted to cooling with liquid nitrogen, as functions of the frequency. For a better readability, the coefficients M_{12} and M_{21} are scaled with the impedance of the duct, $Z = \rho_0 c/S$.

In the low-frequency range, the results show that $\Re(M_{11}) \approx 1$, $\Re(M_{21}Z) \ll 1$, $\Re(M_{12}/Z) \approx -1$ and that $\Re(M_{22}) < 1$ (due to the velocity drop caused by thermoacoustic effects). The results also show that the imaginary parts of those coefficients remain small up to a frequency of around 100 Hz. For higher frequencies, the contribution of the TBT and the heat exchangers to the T-matrix of the TA cell cannot be ignored, which notably impacts the imaginary parts of the T-matrix coefficients. Still, the results show that the short stack approximation of Eq.(6) is a good approximation of the T-matrix of a TA cell up to several tens of hertz.

SUPPLEMENTARY DISCUSSION 4. RIGHT INCIDENT COEFFICIENTS

The measured variations of the absorption coefficient α^- as well as the transmission and reflection coefficients $\mathscr{T}^-$ and $\mathscr{R}^-$, are presented in Fig.6 as functions of the frequency of the incident wave, and for the case of a two-cells configuration.

As for the case of a left-sided incident wave presented in the main document, the experimental results show a good agreement with the theory, especially in the low-frequency range. The results show that when cooling is applied to the right-side of the meshgrids, this leads to an increase of both $\mathscr{T}^-$ and $\mathscr{R}^-$. The results also show that the absorption coefficient α^- appears to be negative in the low frequency range, up to more than 200Hz. This means that the two-port acts as an amplifying unit for a wave incident from the right side (i.e., the sum of transmitted and reflected powers are larger than the incident one). In such a situation, and depending on the boundary conditions applied to both sides of the two-port, this could give rise to the generation of self-sustained acoustic oscillations, as it happens in thermoacoustic engines (which are designed to optimize the conversion from heat into sound).

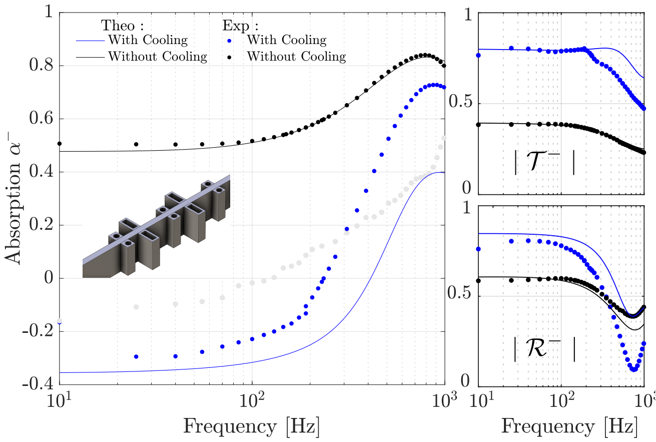

FIG. 6: The absorption coefficient, the transmission and reflection coefficients for a right-sided incident wave, as functions of frequency, and for the two-cells configuration. The solid blue lines correspond to the numerical model for $\eta = 0.42$, while the blue markers ($\circ$) correspond to the experimental data. The black lines and markers represent the theoretical and experimental results without cooling. The soft grey markers correspond to the experimental values of α^- for the case of a single cell with cooling.

SUPPLEMENTARY DISCUSSION 5. SCATTERING OF AN INCIDENT PULSE

To complement the results displayed in the main document, some measurements were also made when the system was excited by pulse signals. Those results are presented here as supplementary information but it is to be mentioned, however, that the distance between microphones could not allow a clear separation of incident and reflected pulses. The signal emitted from the source has a central frequency $f = 300\,\text{Hz}$, and can be emitted either from the left or from the right side of the thermoacoustic absorber, which is here composed of two thermoacoustic cells in series. Fig.7 shows the normalized acoustic pressures measured with microphones Mic. 1 and Mic. 6 (see Fig.2) as functions of the time. The top and bottom plots represent the absorber with or without cooling, respectively. The plots on the left (resp. on the right) correspond to a left-sided (resp. right-sided) incident wave.

The two lower figures clearly show that in the absence of cooling, the two-port acts as a symmetrical and reciprocal absorber, since both figures are almost identical if Mic.1 and Mic.6 are reversed. When cooling is applied to the stack, as shown in the top figures, the non-reciprocity of the two port appears as evident, and the results for a left-sided excitation seem to be consistent with the measurements of the scattering matrix presented in the main document (Fig.4), with some expected reflection and transmission coefficients being around 20% (again, a clear separation of incident and reflected pulses was not achieved here).

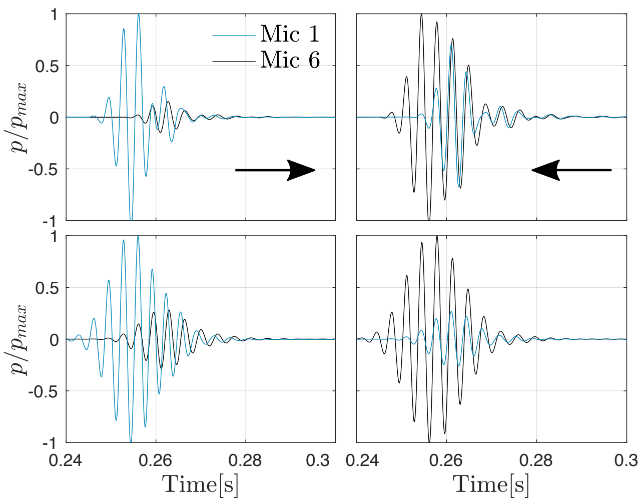

FIG. 7: Measured microphone signals as functions of the time for the two-cells configuration. The incident gaussian pulse has a central frequency $f = 300\,\mathrm{Hz}$, and it is emitted either from the left or from the right, as depicted by the black arrows in the top figures. Top and bottom figures represent the configurations with or without cooling, respectively.

SUPPLEMENTARY REFERENCES

[1] G. W. Swift, "Thermoacoustics: A unifying perspective for some engines and refrigerators," (2003).

[2] N. Rott, "Damped and thermally driven acoustic oscillations in wide and narrow tubes," Zeitschrift für angewandte Mathematik und Physik ZAMP **20**, 230–243 (1969).

[3] M. Åbom, "Measurement of the scattering-matrix of acoustical two-ports," Mechanical systems and signal processing **5**, 89–104 (1991).

[4] H. Bodén and M. Åbom, "Influence of errors on the two-microphone method for measuring acoustic properties in ducts," The Journal of the Acoustical Society of America **79**, 541–549 (1986).

[5] J.-P. Dalmont, J. Kergomard, and X. Meynial, "Réalisation d'une terminaison anéchoïque pour un tuyau sonore aux basses fréquences," Comptes rendus de l'Académie des sciences. Série 2, Mécanique, Physique, Chimie, Sciences de l'univers, Sciences de la Terre **309**, 453–458 (1989).

[6] S. W. Rienstra and A. Hirschberg, "An introduction to acoustics," Eindhoven University of Technology **18**, 19 (2004).
